# Value of ultrasound assessment for traumatic nerve injury of the upper limb

**DOI:** 10.1007/s40477-022-00756-2

**Published:** 2022-12-22

**Authors:** Islam Elhefnawi Elshewi, Mona Mohammed Fatouh, Rahma Nour Eldin Saad Mohamed, Mye Ali Basheer, Nevien Ezzat El Liethy, Hoda Magdy Abbas

**Affiliations:** 1 Radiology Department, Faculty of Medicine, Cairo University, Cairo, Egypt; 2 Special Medicine Department, Faculty of Medicine, Cairo University, Cairo, Egypt

**Keywords:** Ultrasound, Peripheral nerve, Trauma

## Abstract

**Aim of work:**

The type of traumatic peripheral nerve injury is a key factor for determining optimal treatment. Proper assessment of peripheral nerve injury facilitates appropriate treatment, significantly affects prognosis, and reduces disabilities. This study evaluated ultrasonography (US) to assess upper limb traumatic nerve injuries and compared the US with electrodiagnostic studies as the gold standard.

**Materials and Methods:**

Participants were 69 adults (57 [83%] men, 12 [17%] women; mean age 36.3 ± 13.5 years) with a total of 96 peripheral nerve injuries (duration of 1 month–3 years). High-frequency US examinations and electro-physiologic studies confirmed upper limb peripheral nerve injury.

**Results:**

Nerve discontinuation was diagnosed in 15 (15.6%) nerves; the cross-sectional area was increased in 33 (34.4%) nerves. Of 96 injuries, 54 (56.3%) were median, 24 (25%) were ulnar, and 18 (18.8%) were radial nerves. No statistically significant difference was found between US and electro-physiologic studies for nerve injury diagnosis (p = 0.054).

**Conclusion:**

No significant differences were found between US and electro-physiologic studies for diagnosis of nerve injuries; however, US was valuable to assess surrounding tissue and supplied muscles. The capabilities to detect nerve injury and associated distal muscular, vascular, and other regional structures position the US as a complementary diagnostic tool.

## Introduction

Peripheral nervous system trauma is rare, but this injury can have a significant negative influence on a patient's health and functional capacities. The overall incidence is 2–3% of patients who visit major trauma centers worldwide, or 13–23 per 100,000 people annually. The most frequent cause of peripheral nerve injuries (PNIs) during calm times is motor vehicle collisions, whereas less prevalent causes are penetrating trauma, falls and industrial accidents [1]. The risk of PNI increases to about 5% when plexus and root injuries are taken into account. Iatrogenic injury is also common. For example, cervical lymph node excisions have a 3–6% risk of PNI to the auxiliary nerve, and radial nerve injury from humeral shaft osteosynthesis for fracture stabilization has a 5–15% risk of PNI, in addition to the inherent risk of the fracture [1].

Specific mechanisms of trauma increase the risk of specific nerve injury. To determine which nerves may be impacted, knowledge of the precise trauma mechanism is necessary. This knowledge should take precedence over the conventional neurologic methodology when localizing the peripheral nervous system disease during workup [1]. Most PNIs in the upper limb are the ulnar, median, and radial nerves, whereas PNIs in the lower limb are most often the sciatic nerve, followed by the peroneal nerve, and, less frequently, the tibial and femoral nerves [2].

Despite the fact that localization of PNIs is clinically possible accurate localization of the injury is challenging, especially when many nerves are involved. Electrophysiological studies are useful to detect the injury severity and location. However, because the most accurate diagnostic information from these studies is only accessible after 2 weeks when Wallerian degeneration is fully developed, electro-diagnostic techniques are not well suited for the acute phase and are inherently painful. In addition, in individuals with a total loss of nerve conduction, electro-diagnostic testing cannot reveal the precise location of the lesion site. Furthermore, these studies cannot display the morphologic changes of the injured nerve [3, 4].

Compared with electro-diagnostic tests, ultrasonography (US) can accurately and painlessly provide this information. Within the whole clinical workup for PNI, the US has a complementary and expanding role. It not only offers a quick and affordable method to probe extensive stretches of peripheral nerves, but it also has unique benefits based on its dynamic, real-time nature and its small number of clinical limitations and contraindications. Thanks to developments in high-frequency transducers and post-processing methods, the function of the US is expanding [5].

When using the US, it is essential to determine the following factors: whether the nerve is still continuous; the size and position of any gaps in the nerve course; the presence of any focal neuromas or extra nerve damage sites, such as tandem lesions; the presence of any foreign bodies; the degree of neighboring scar tissue; and the condition of surrounding tissues and structures, such as tendons, arteries, and bones [4]. As an advantageous procedure in the evaluation of peripheral entrapment syndromes, the US can determine if the nerves are compressed, tethered, or hypermobile with respect to adjacent structures. Shifting transducer pressure may have an impact on sono-palpation, which can be used to demonstrate that the patient’s condition is neuropathic pain, find the location of the peripheral nerves responsible for the symptoms, and identify the exact site that consistently triggers the patient's symptoms, often identifying the responsible basic pathology. Because the whole longitudinal course nerve is typically evaluated in a single examination, it is possible to detect multi-segmental disease with cost-effectiveness [5].

Our study will evaluate the value of the US in the assessment of traumatic nerve injuries of the upper limb compared with the role of electro-diagnostic studies as the current gold standard diagnostic modality.

## Materials and methods

From December 2021 to June 2022, adult patients with a history of PNIs were recruited from the clinical neurophysiology unit at our university after undergoing physical examination by an orthopedic surgeon subspecialized with 10 years of experience. The study participants were 69 patients with 96 nerve injuries, including 57 (83%) men and 12 (17%) women, with a mean age of 36.3 ± 13.5 years. All of the study participants gave informed written consent to the study.

Inclusion criteria were a clinical picture of PNI in the upper limb, with a duration of 1 month up to 3 years; and surgical exploration of the PNI. Exclusion criteria were diabetes mellitus, polyneuropathies, renal or hepatic disorders, or radiculopathy (clinical or electrophysiological in the segment supplying the injured nerve); and refusal to participate in the study. All patients underwent thorough history-taking and clinical examination. These patient history data were collected: date of injury date; onset of neurologic manifestations, such as weakness, numbness, and loss of sensation; and history of surgical intervention for the injury.

### Clinical examinations

The following clinical examinations were performed for all patients.

### Electrophysiological studies

The electrophysiological research was performed using the Neuropak MEB-9200G/K EP/EMG measurement device (Neuropak M1, version 08.1, Nihon Kohden, Tokyo, Japan). Standard motor nerve conduction studies (NCS) were performed to record the nerve damage for these sites and data: abductor pollicis brevis; abductor digiti minimi; wrist; below and above the elbow stimulation for the ulnar nerve, extensor indices properties; and forearm and elbow stimulation for the radial nerve. The second digit (antidromic stimulation at the wrist for the median nerve), the fifth digit (antidromic stimulation at the wrist for the ulnar nerve), and the anatomical snuff box were recorded as part of routine sensory NCS (antidromic stimulation at the wrist for the superficial radial nerve).

### Needle electromyography

To determine the level of injury needle electromyography (EMG) was performed for the muscles supplied by the injured nerve using concentric needles first distally and then proximally. To rule out numerous sites of nerve damage an EMG investigation was also performed for neighboring muscles.

### Sonography

Conventional high-resolution US in the brightness mode was performed for all patients using Aplio 500 (Toshiba, Tokyo, Japan) with a linear probe of 714 MHz frequency. The US examinations were performed by a radiologist with 15 years of experience and who was blinded to the results of the electrodiagnostic tests and clinical Examinations. Depending on the patient's overall condition, patients were either examined while lying flat on their back or comfortably sitting facing the radiologist. The elbow was supported and flexed, and the forearm was extended when the affected nerve was examined at the forearm. To examine the hand, the wrist was held steady and positioned slightly hyperextended. A generous amount of gel was used to improve contact between the probe and the skin's surface; in cases of painful neuromas, little pressure was used. Using sterile gel and taking proper antiseptic precautions, such as using disposable non-sterile gloves and cleaning the US machine and transducer before and after scanning is advised for susceptible patients or while scanning in wound areas.

To compare the healthy and injured nerves, a US examination was also performed on the healthy unaffected side. Using a recognized anatomic landmark, the nerves were identified sonographically. Once identified, the nerve was tracked proximally and distally while remaining in the center of the US image along its short axis. The area of interest was then positioned properly focused during US inspection using transverse and longitudinal scanning planes.

### Image interpretation

To interpret the images, the following steps were taken and factors were assessed:

1. Identification of the injured nerve lesion site by elicitation of the Tinel sign during US examination because it can help identify the nerve lesion site while using sonopalpation, or the compression of the US probe.
2. Testing for nerve continuity, which includes determining whether the nerve's fascicle is swollen and hypoechoic, whether it lacks a typical fascicular pattern, and whether a neuronal stump is present.
3. Degree of nerve injury, determined to be partial or complete based on the Sunderland classification of PNI [6], as follows:
  - Sunderland grade 1: No pathologic findings on the US are visualized or only mild swelling of the nerve with an intact fascicular pattern.
  - Sunderland grade 2: Enlarged cross-sectional area (CSA) of affected fascicles and nerves at the lesion site because of axonal swelling and edema are evident.
  - Sunderland grades 3 and 4: Lesion shows a loss of the normal nerve architecture and echotexture with a disruption of the fascicular pattern and often sizable hypoechogenic enlargement of the nerve.
  - Sunderland grade 5: Lesion shows transection of the nerve with a loss of nerve continuity.
4. Amount of nerve retraction and the gap after complete nerve transaction.
5. Nerve thickening, by measuring the CSA of the injured nerve within the hyperechoic epineurial rim of the nerve and the transducer perpendicular to the nerve.
6. Bony fragments pressing against the nerve near the site of the bone fracture.
7. Proximal terminal neuroma, characterized by uniform texture, hypoechoic echogenicity, and concentric swelling at the terminal end of a transected nerve, with measurement of its size by placing several diameter markers proximal to, at, and caudal to the lesion site.
8. Neuroma in-continuity, by determining if it is intact and manifests as a broadening of the nerve's contour in a nodular shape.
9. Perineural fibrosis, vascular injury, and retained foreign bodies, by evaluating for their presence.
10. Muscular injury, recognized in this study as an increase in echogenicity of the supplied muscle and/or reduced girth (atrophic changes).
11. Doppler brightness mode with color or power Doppler to quantify blood flow for both intraneural and epineural compartments.

### Statistical analysis

Statistical analysis was conducted using SPSS (version 22, IBM, Armonk, NY, USA). Quantitative variables were presented as mean ± standard deviation and range, and qualitative variables were presented as frequency and percentages. Paired comparison of diagnostic tests was conducted using the McNamara test, comparison between proportions was conducted using the chi-squared test. Sensitivity, specificity, positive predictive value (PPV), negative predictive value (NPP), and overall diagnostic accuracy were calculated using a 2 × 2 contingency table. Any value of *p* < 0.05 was considered significant.

## Results

A total of 69 patients with 96 nerve injuries were eligible for inclusion in the final analysis, with 57 (83%) men and 12 (17%) women. The mean age was 36.3 ± 13.5 years. Some patients had more than one injured nerve.

**Fig. 1:**
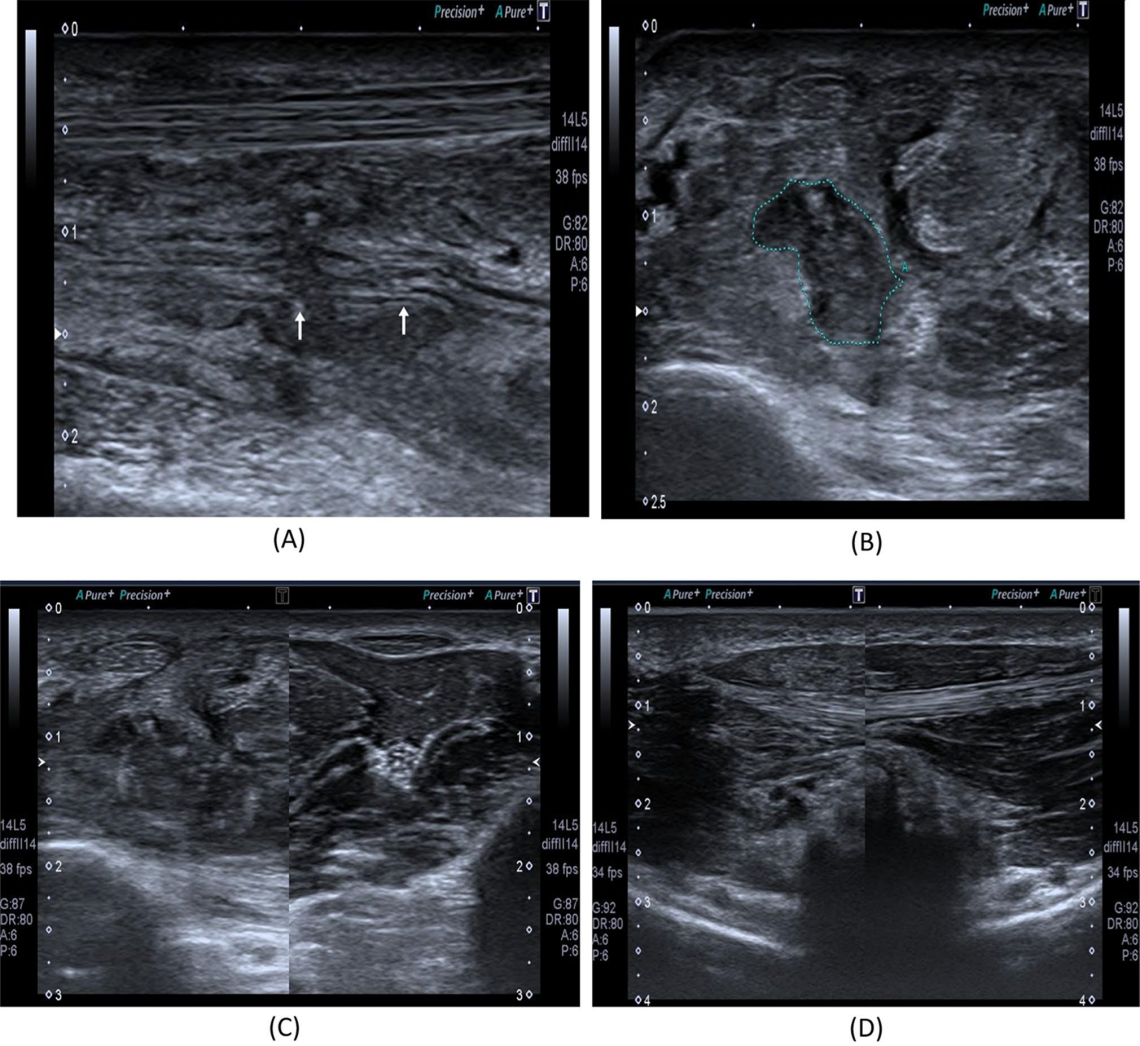
An 18-year- old male with a history of a cut wound of the right forearm since September 2021 and tendon repair, sensory loss of the skin on the radial aspect of the palm with skin ulceration. **A** B-mode image shows a discontinuous right median nerve at the mid-forearm with related hypoechoic tissue suggesting complete nerve injury proved by NCS. **B** B-mode image shows a transverse axis view at the site of the lesion showing a hypoechoic area with non-visualized nerve tissue. **C** B-mode image shows a comparison between the right and left mid-forearm muscles. The right side shows an abnormal architecture of the FDS and FDP muscles with increased echotexture as compared to the left normal side. **D** B-mode image shows a comparison between the right (left image) and left (right image) thenar muscles. The right side mildly increased echotexture as compared to the left normal side with rather preserved girth

**Fig. 2:**
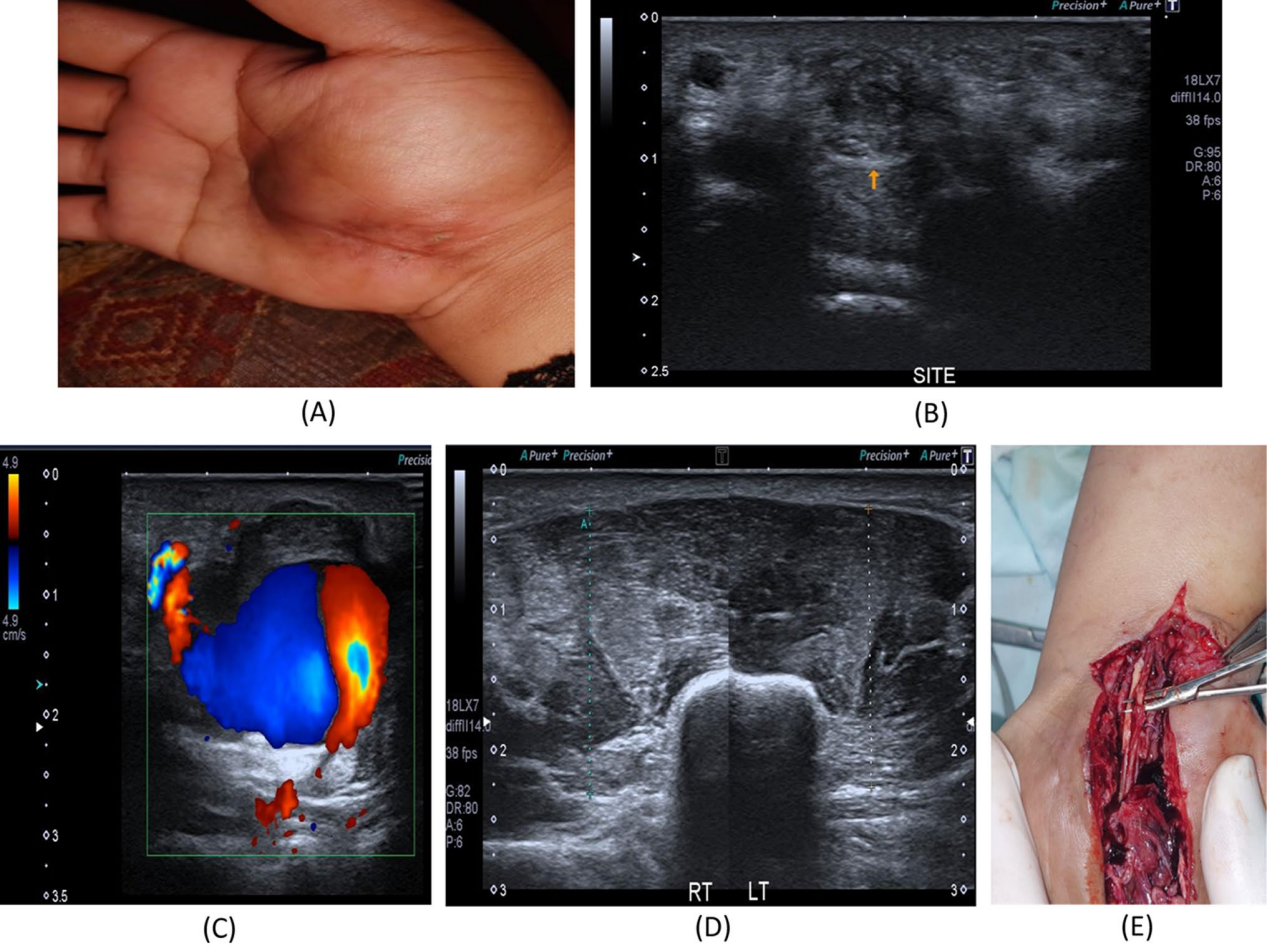
26-year- old female with a history of right distal radial fracture in July 2021 treated, After the cast removal, numbness and tingling along the radial aspect of the palm with loss of sensation of the middle finger and newly developed progressive increased thenar swelling. **A** Patient’s hand with prominent swelling of the thenar eminence and mid-palmar incision scar. **B** B-mode image, transverse axis, reveals loss of normal sonographic appearance of the left median nerve at the carpal tunnel with related hypoechoic superficial scar tissue, suggesting partial nerve injury. **C** Color Doppler mode image reveals pseudo-aneurysm; Pepsi sign with an area of luminal thrombosis. **D** The B-mode image of the thenar muscles, increased echogenicity of the left thenar muscles as compared to the right side with rather preserved girth. **E** Intraoperative image revealed complete injury of two of the sensory branches of the median nerve

**Fig. 3:**
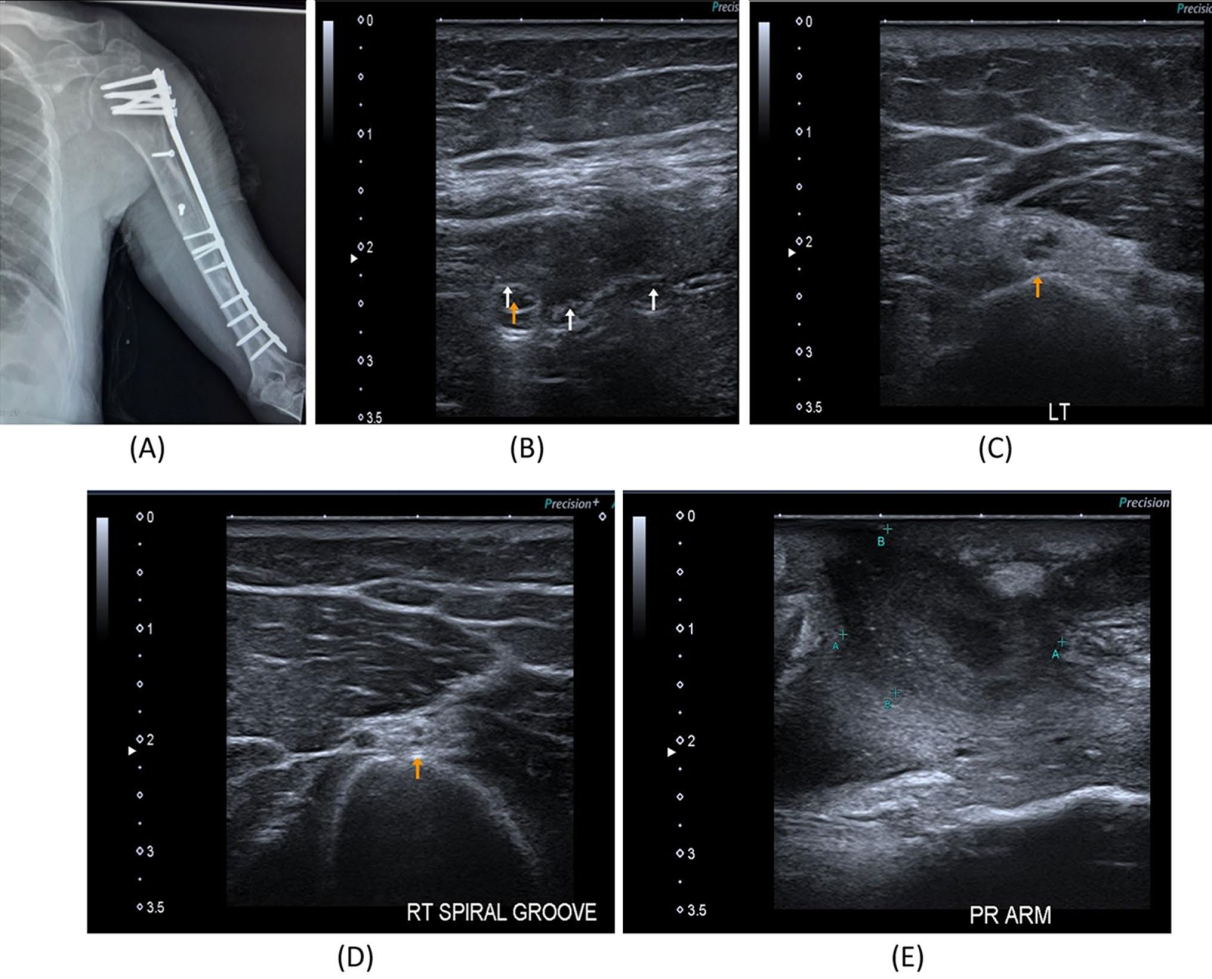
A 35-year- old male with a history of left humeral fracture with internal fixation with plate and screws since July 2021 with a weak extension of the elbow. **A** Digital radiography image of the left arm showing internal fixation with plate and screws. **B** B-mode image, hypo-echogenicity saw along the course of the radial nerve within the spiral groove intimately related to applied screws. **C** B-mode image, transverse axis, thickened left radial nerve in the spiral groove of the humerus, suggested axonal injury proved by NCS. **D** B-mode image, transverse axis of the right normal radial nerve within the spiral groove of the humerus. **E** B-mode image, turbid collection noted the lateral aspect of the axilla

**Fig. 4:**
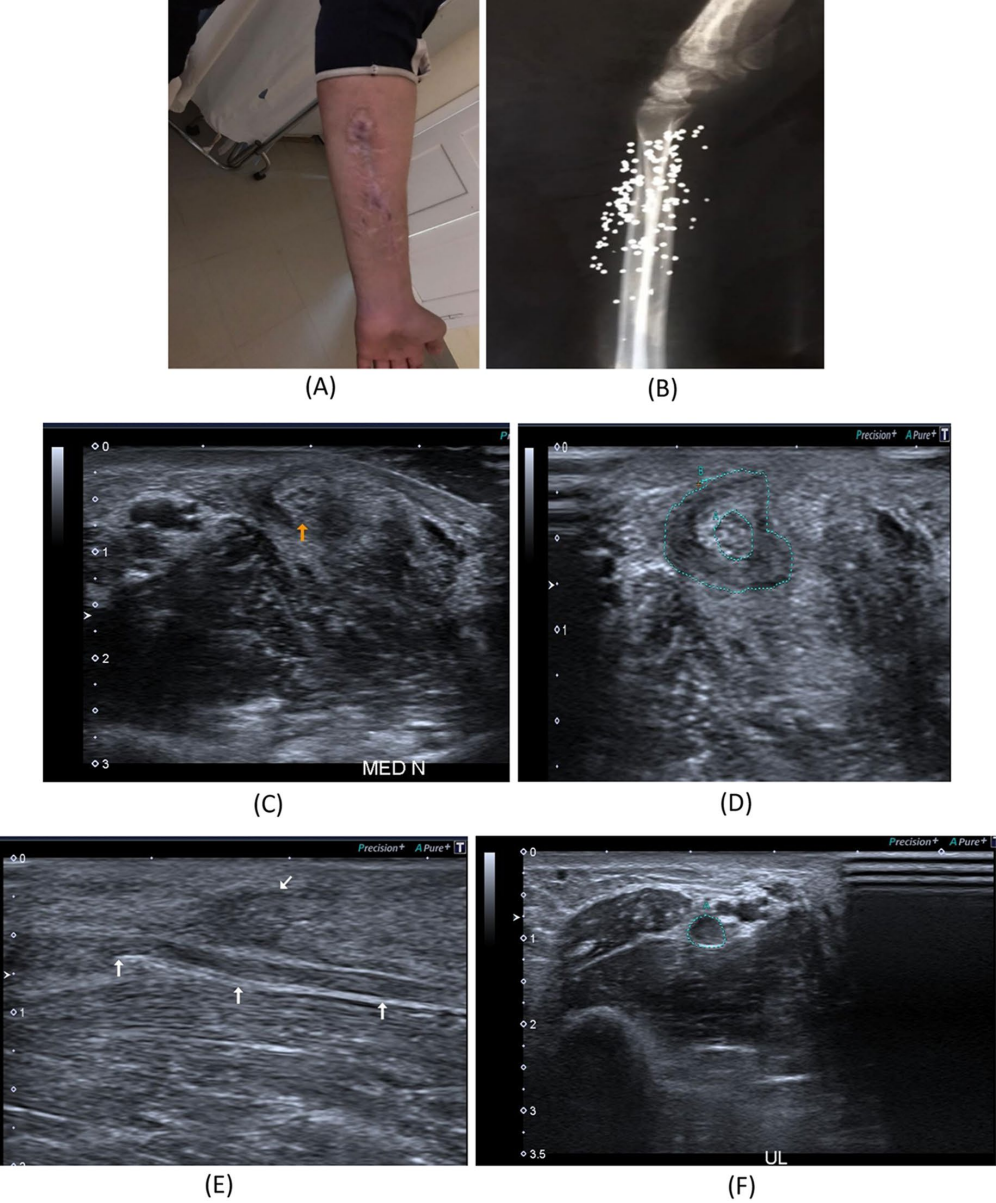

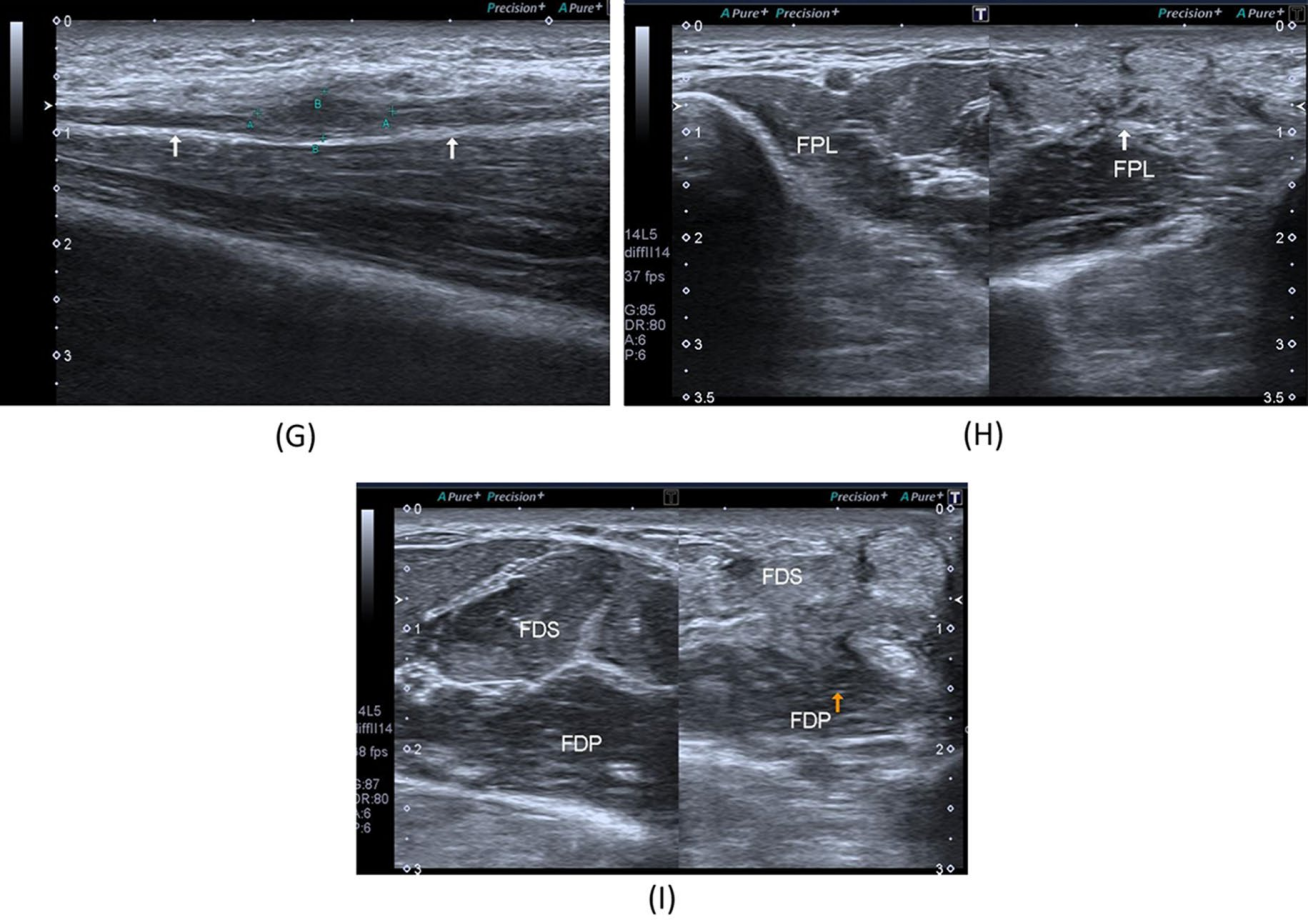
A 28-year- old male patient presenting with history gunshot injury to the left forearm since June 2021. He underwent flexor muscles and tendons in addition to median nerve repair. **A** Patient’s forearm showing scar tissue. **B** X-ray image of the left forearm multiple retained bullets. **C**, **D** B-mode image, transverse axis, shows thickened median nerve(arrow) at proximal forearm with circumferential hypoechoic lesion. **E** B-mode image, longitudinal axis, shows intact epineurum of the median nerve(arrows) at proximal forearm with superficially related hypoechoic lesion (oblique arrow). **F** B-mode image, transverse axis, shows thickened hypoechoic ulnar nerve at proximal forearm. **G** B-mode image, longitudinal axis, shows thick hypoechoic swelling of the left ulnar nerve with loss of fascicular architecture. **H** B-mode image, transverse axis, abnormal architecture of the left FPL muscle with increased echogenicity and reduced girth as compared to the normal right side. **I** B-mode image, transverse axis, abnormal architecture of the left FDS muscle with increased echogenicity and reduced girth as compared to the normal right side

**Fig. 5:**
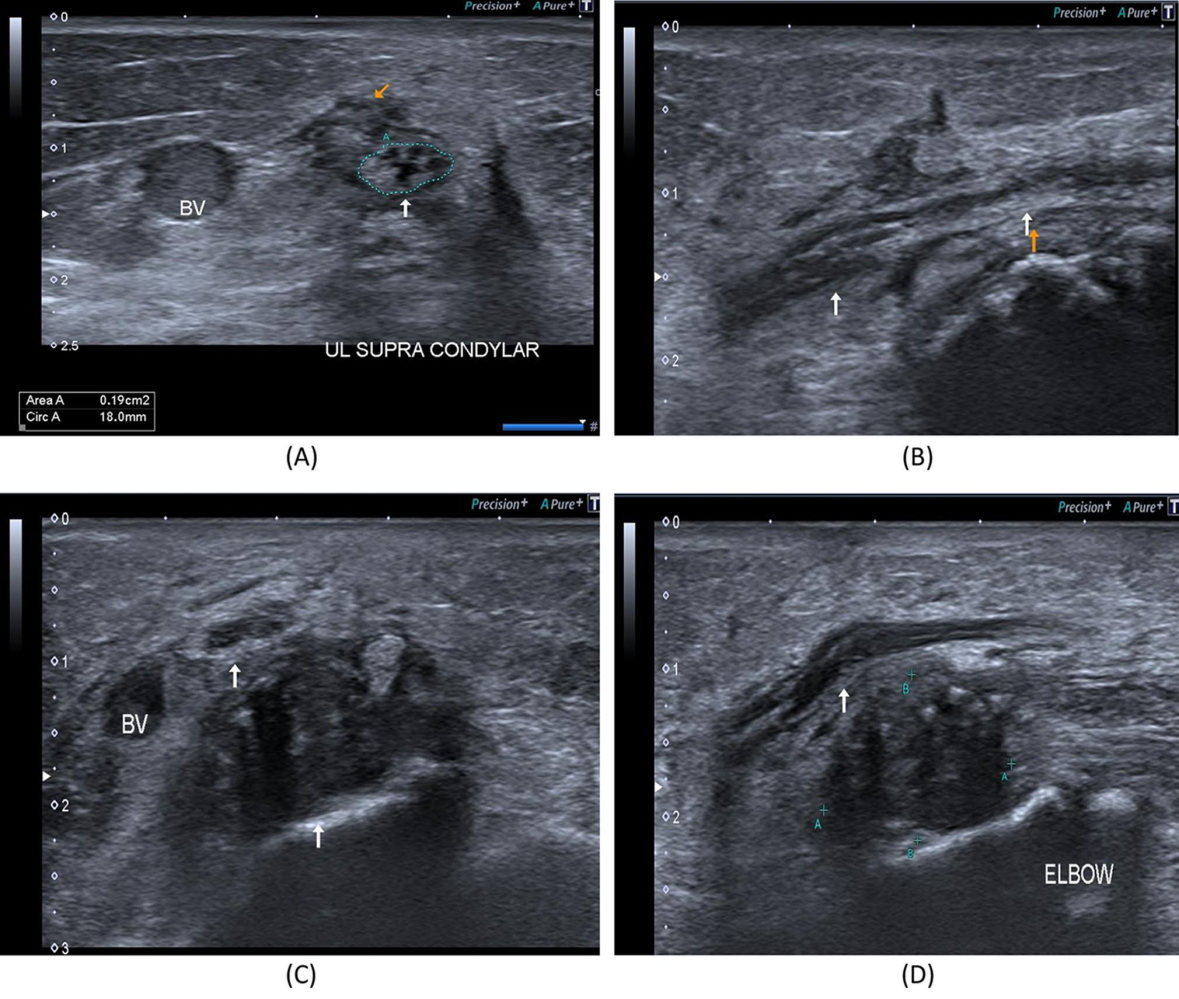
A 33-year- old female patient presenting with history of right medial epicondyle fracture which was treated by applying a cast in June 2021. After removal of the cast, anterior transposition and neurolysis of the ulnar nerve was done. **A** B-mode image, transverse axis, shows thickened right ulnar nerve, CSA 19mm2, just proximal to the medial epicondyle with intimately related hypoechoic scar tissue, BV; basilic vein. **B** B-mode image, longitudinal axis, shows focal interruption of the epineurium of the right ulnar nerve (arrows) with related hypoechoic scar tissue. **C** B-mode image, transverse axis, shows thickened right ulnar nerve (superior arrow), at the level of the medial epicondyle with related hypoechoic synovial thickening containing echogenic foci of bone fragments (inferior arrow). **D** B-mode image, longitudinal axis, shows indented right ulnar nerve at the level of the medial epicondyle with related hypoechoic synovial thickening containing echogenic foci of bone fragments

**Fig. 6:**
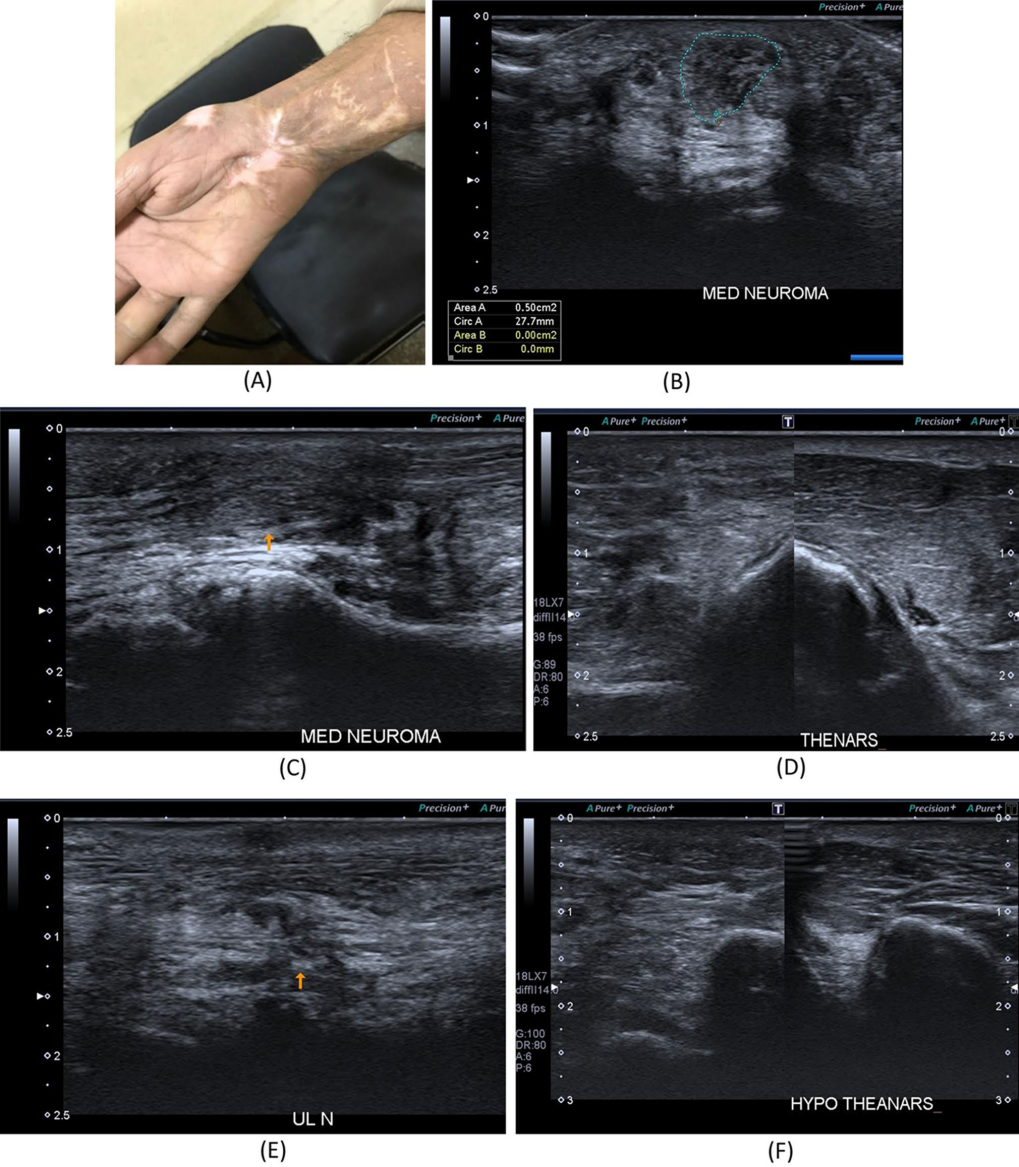
A 25-year- old male patient presenting with history of left wrist compression/degloving injury in 2020. He underwent tendon repair surgery. **A** Patient’s hand. **B** B-mode image, transverse axis, reveals thick hypoechoic swelling of the left median nerve with loss of fascicular architecture. **C** B-mode image, longitudinal axis, thickened, hypoechoic focal area with loss of fascicular architecture of the median nerve at the site of injury. **D** B-mode image of the thenar muscles, reveal loss of normal sonographic appearance of the left side muscles with increased echogenicity as compared to the right side. **E** B-mode image, longitudinal axis, shows discontinuous, disorganized left ulnar nerve at the distal forearm with related hypoechoic scar tissue formation. **F** B-mode image of the hypothenar muscles, reveal loss of normal sonographic appearance of the left side muscles with increased echogenicity as compared to the right side

**Fig. 7:**
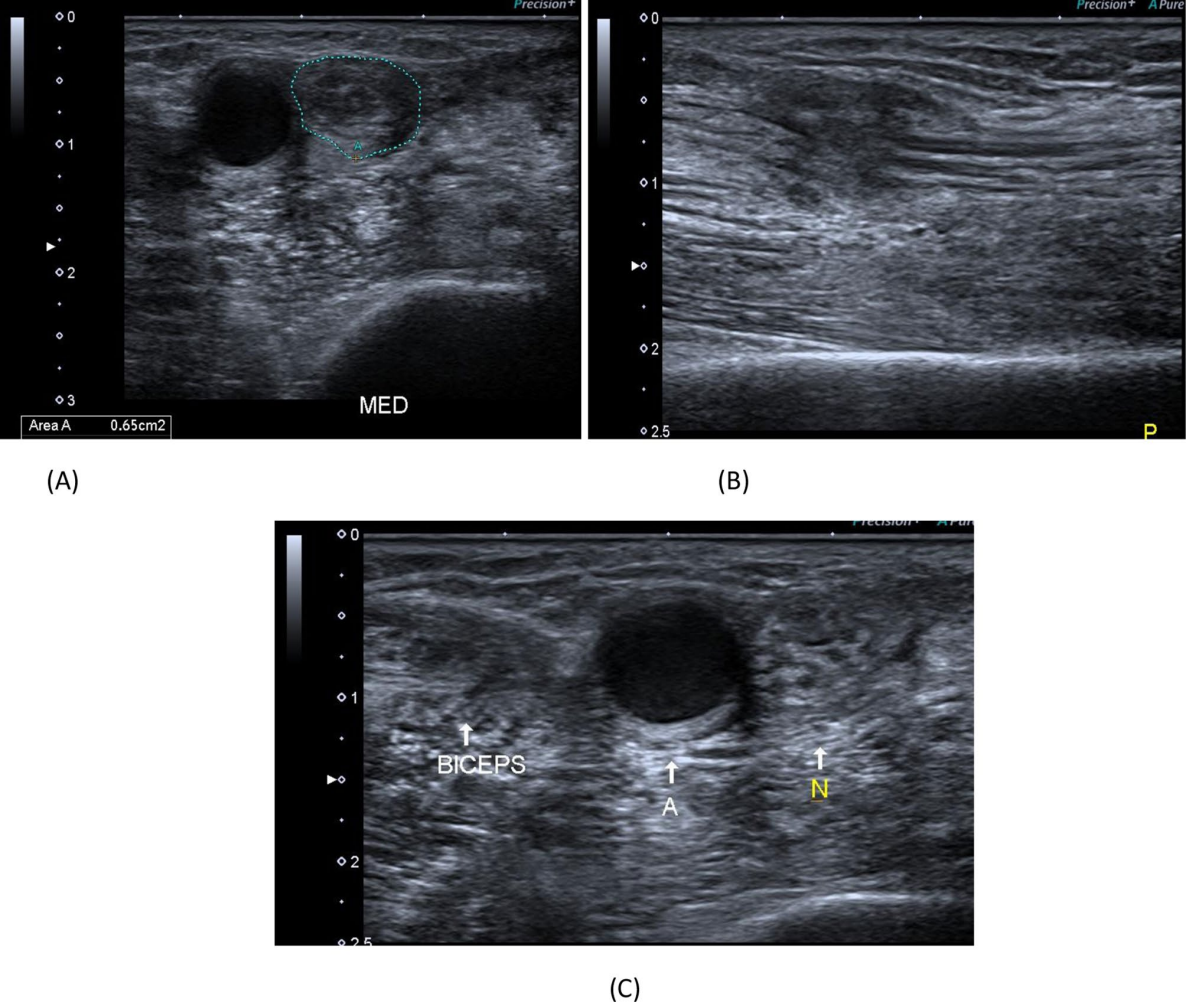
A 40-year- old male patient presenting with history of cut wound of the right arm just proximal to the elbow crease since September 2021. He gave history of operative repair of brachial artery injury. **A** B-mode image, transverse axis, shows abnormal architecture of the right median nerve at the distal arm with loss of the normal honeycomb appearance and related hypoechoic scar tissue formation. **B** B-mode image, longitudinal axis, shows discontinuous right median nerve at the distal arm with related hypoechoic scar tissue formation. **C** B-mode image, transverse axis, shows abnormal architecture of the right median nerve(N) at the distal arm with loss of the normal honeycomb appearance and related hypoechoic scar tissue formation, aneurysmal dilatation of the brachial artery (**A**) and distorted biceps muscle (BICEPS)

The mode of trauma was crush injury (27 cases), cut wounds (27 cases), compression (9 cases), and traction (6 cases). Of the 96 injured nerves, 27 were on the right side and 69 (71.9%) were on the left side. The injury level was at the arm in 39 nerves, followed by the forearm (33 nerves), and 24 injuries were at the hand. Injured nerves were mainly median nerve in 54 (56.3%) cases, ulnar in 24 (25%) cases, and radial nerve in 18 (18.8%) cases. Table 1 presents these data. As shown in Table 2, US findings showed 27 (28.1%) intact nerves, neuroma in 21 (21.9%) nerves (Figs. 1, 2, 3), nerve discontinuation in 15 (15.6%) (Figs. 4 and 5) nerves and an increase of CSA of the injured nerve in 33 nerves (34.4%) (Figs. 6 and 7).

**Table 1:** Clinical characteristics of trauma

	Count	Column *N* %	
Mode	Cut wound	27	28.1
Crush	27		28.1
Traction	6		6.25
Compression	9		9.3
Side	Right	27	71.8
Left	23		71.9
Site	Arm	39	40.6
Forearm	33		34.3
Hand	24		25.0
Nerve	Median	54	56.3
Ulnar	24		25.0
Radial	18		18.8

**Table 2:** Ultrasound findings among the included patients

	Count	Column *N* %
US findings		
Intact	27	28.1
Neuroma	21	21.9
Discontinuous	15	15.6
Increased CSA	33	34.4

Increased echogenicity of supplied muscle was observed in 81 (84.4%) nerve injuries. In addition, 69 (71.9%) injured nerves had a reduced muscle girth, as shown in Table 3. Other injuries were noted for 48 patients who had associated fibrosis 9 patients who had associated vascular injury 24 patients who had an orthopedic injury and 9 patients who reported other types of injuries. Also, 30 patients had muscle injuries. These findings are presented in Table 4.

**Table 3:** Ultrasound findings regarding supplied muscles

	Count	%
Echogenicity		
Normal	15	15.6
Increased	81	84.4
Muscle Grith		
Normal	27	28.1
Reduced	69	71.9

**Table 4:** Associated ultrasound findings

	Count	Column *N* %
Fibrosis		
Absent	48	50.0
Present	48	50.0
Vascular		
Absent	87	90.6
Present	9	9.4
Orthopedic		
Absent	72	62.5
Present	24	37.5
Others		
Absent	87	87.5
Present	9	12.5
Muscle injury		
Absent	66	59.4
Present	30	40.6

The 27 cases with intact nerves shown in the US were diagnosed by electrophysiological studies with mild to moderate nerve injuries. These studies showed that the severity of nerve injury was mild in 18 (18.8%) nerves, moderate in 45 (46.9%) nerves, and severe in 21 (21.9%) nerves, with complete injury in 9 nerves (9.4%), whereas 3 nerves were intact. The axonal injury was reported by NCS in 96 (100%) nerves. These data are presented in Table 5.

**Table 5:** Electrophysiological studies among the included patients

	Count	Column *N* %
Severity		
Intact	3	3.1
Mild	18	18.8
Moderate	45	46.9
Severe	21	21.9
Complete injury	9	9.4
Axonal injury	96	100

No statistically significant difference was found between US and NCS in terms of the diagnosis of nerve injuries (*p* = 0.054; sensitivity 100%; specificity 95.8%; NNP 100%; PPV 95.8%; overall diagnostic accuracy 97.8%). Tables 6 and 7 present the statistical data.

**Table 6:** Paired comparison of ultrasound and nerve conduction studies of the traumatized nerve

	Nerve conduction study				*p* value
	Negative		Positive		
	Count	Row *N* %	Count	Row *N* %	
Ultrasound					
Negative	1	11.1%	8	88.9%	0.054
Positive	0	0	78	100%

**Table 7:** Diagnostic indices of ultrasound compared to nerve conduction study

Statistic	Value (%)	95% CI
Sensitivity	100.00	85.18–100.00%
Specificity	95.83	78.88–99.89%
Positive predictive value	95.83	77.15–99.37%
Negative predictive value	100.00	
Accuracy	97.87	88.71–99.95%

## Discussion

Injury to the peripheral nerves causes significant disability to patients around the world [1]. The common causes are motor vehicle collisions, falls, industrial accidents, domestic accidents and penetrating trauma [7]. Electrodiagnostic investigations are the gold standard for diagnosis of traumatic PNIs. These studies can be used to locate lesions, diagnose the underlying pathology, assess the extent and severity of axonal degeneration, and monitor recovery [8]. However, electrodiagnosis has a limited capacity to pinpoint the exact site and degree of nerve damage in certain situations, notably in the early post-injury period, because some electrodiagnostic alterations take time to manifest [9]. These studies also are restricted in their capability to detect morphological changes associated with a specific type of nerve injury [5].

In comparison to electrodiagnostic examinations, the US can reliably offer this information in a painless manner. In the overall clinical workup, the US plays a complementary, growing role. Not only does it provide a cost-effective and time-efficient method of interrogating long segments of peripheral nerves, but it also has special advantages in terms of its dynamic, real-time nature, as well as a limited number of clinical contraindications and limitations. The role of the US is expanding thanks to advancements in high-frequency transducers and post-processing techniques [5].

The present study aimed to evaluate the role of high-frequency US in the diagnosis of traumatic PNIs and to compare US findings with the electrodiagnostic findings. Therefore, we conducted a cross-sectional study that included 69 patients with 96 nerve injuries. These cases include 57 (83%) men and 12 (17%) women, with a mean age of 36.3 ± 13.5 years old. These findings are consistent with the evidence in the literature from Ciaramitaroi [10] and Bösch [11], which found that PNIs more often occur in male versus female individuals, and the most common age group to be affected by PNIs is patients aged 30–40 years.

Kamble [12] reported that among 1–2% of individuals with PNIs associated with central nervous system damage, 60% of cases were spinal injuries, fractures, and dislocation of adjacent bones. About 10–34% of patients hospitalized in the rehabilitation center have related nerve damage. Also, early diagnosis and care are vital to enhancing the functional prognosis in these individuals; consequently, it is necessary to identify the related nerve damage.

A study by Martins [13] showed that the degree and type of nerve injury involved in traumatic neuropathy must be determined because therapy for different degrees and types of traumatic neuropathy may vary. In cases of severe nerve damage (Sunderland classification group 5, neurotmesis), surgical intervention is essential, and it may also be required in cases with Sunderland groups 3 and 4. In the current study, the most common cause of PNI was crush injury and cut wounds in 54 of 69 cases, followed by the other types of injuries. In contrast, Uzun [8] and Elfayoumy [14] reported that cut injury by sharp objects was the most common cause of PNI. The site of laterality of the injured nerves in our study was more often on the left side (71.9%). Ferrante [15] reported that the most frequent nerve injury in the upper limbs is the radial nerve, followed by the median and ulnar nerves, whereas our study showed the median nerve was the nerve most often injured (56.3%), followed by the ulnar (25%) and radial (18.8%) nerves.

Koenig [16] divided the nerve injury findings in the US into five groups: normal, epineural fibrosis, intraneural fibrosis, neuroma/partial neuroma, and transacExtremite [17] examined 36 patients with nerve injuries in the upper limbs and showed the excellent capability of US in determining the type of injury and detection of proximal and distal nerve stump, foreign particles, stump neuroma, and perilesional excessive scar tissue formation. In our study, neuroma was detected in 21 (21.9%) nerves, and 33 nerves (34.4%) showed an increase in CSA of the injured nerve. In addition, 48 patients had associated fibrosis. These findings almost paralleled those of the study by Elfayoumy [14], who divided high-resolution US findings into three groups: fusiform in shape (increased CSA) (36.7%), fibrosis (36.7%), and neuroma (26.6%).

In the present study, no statistically significant difference was revealed between US and NCS for diagnosis of PNI (p = 0.054; sensitivity 100%; specificity 95.8%; NNP 100%; PPV 95.8%; overall diagnostic accuracy 97.8%). These findings have high agreement with those by Assy [18], who assessed 30 patients with suspected traumatic nerve injury by higher resolution US and NCS. Their results for detection on US of neuroma, and local thickening were 100% and 83.3% sensitivity, 94.4% and 100% specificity, 96.7% and 90% accuracy, 92.8% and 100% PPV, and both 100% NPV, respectively. Another study by Cartwright [19] in cadaveric nerves confirmed that US can detect transactions with 89% sensitivity and 95% specificity. Zhu [20] compared the severity of nerve injuries in US with surgical findings and reported the sensitivity of US was 93.2%.

The limitations of this study are the relatively small number of patients and the limited availability of postoperative findings in all patients.

## Conclusion

Results showed that the US can be used efficiently as a complementary diagnostic tool in the assessment of PNIs based on its capability not only to detect the type of injury and morphological abnormalities of affected nerves, but because it also allows proper assessment of the surrounding tissue, including swelling, scar tissue, and neuroma formation, thus altering management decisions as needed.

## Data Availability

The datasets used and/or analyzed during the current study are available from the corresponding author on reasonable request.

## Abbreviations

B-mode: Brightness mode
CI: Confidence interval
CSA: Cross-sectional area
CTS: Carpal tunnel syndrome
CMAPs: Compound motor action potentials
EDX: Electrodiagnostic studies
EMG: Electromyography
FCU: Flexor carpi ulnaris
FDP: Flexor digitorum profundus
FPL: Flexor pollicis longus
ME: Medial epicondyle
LS: Longitudinal section
MUAPs: Motor unit action potentials
NCS: Nerve conduction study
NCV: Nerve conduction velocity
NMJ: Neuromuscular junction
NPP: Negative predictive value
PPV: Positive predictive value
TS: Transverse section
RNS: Repetitive nerve stimulation
SD: Standard deviation
SNAPs: Sensory nerve action potentials
US: Ultrasound
